# 

**Published:** 1888-04-21

**Authors:** 


### Special Notice to Newsagents, Advertisers, and the Trade.

**CHANGE OF OFFICE AND PUBLISHER.**

Notice is hereby given that the Office of The Hospital will henceforth be at 38, Old Jewry (Cheapside end), E.C., to which address all communications should be sent. Mr. A. Doig has been appointed Advertising Agent, and Mr. J. H. S. Hanning, Manager. All Cheques and Post Office Orders should be made payable to J. H. S. Hanning, 38, Old Jewry, E.C. crossed Lloyds, Barnetts, and Bosanquets Bank (Limited).

### To Secretaries and Matrons.

The Editor will be glad to have a copy of the Regulations, and also of every Report issued from the Press of Asylums, Hospitals, Convalescent and Nursing Institutions, as well as those of other Charities, so soon as they are published, and an early copy in duplicate of all appeals for funds. Notices of Annual and other Meetings, Festival Dinners, etc., will be welcomed. All such communications should be addressed to *The Editor, The Lodge, Porchester Square, London, W.* Any superintendent, secretary, matron, or other chief official who will regularly send such documents and information will be registered to receive free of cost a cover for binding The Hospital in half-yearly volumes on sending name and address to the publisher.

### DIFFICULT CASES.

[Replies endorsed "Difficult Cases," should be addressed to the Editor.]

### MAIMED BY NATURE.

Will some reader of The Hospital take compassion on the following case? A young man aged 20, in the Whitchurch Union House, Hants, has been denied by nature the handles of his body,—the arms, having in their place only small apologies for them. He can write well, can dress himself, is very strong, can carry great weights, and is very steady and good-looking. If some kind friend would help him to a place as messenger, or gate-keeper, a great charity would be done. He is anxious to work for his own livelihood.

F. W. Hight,

Chairman, Whitchurch Union, Hants.

---

We have received some "Lines to a Deserted Study," by S. Weir Mitchell, from which we quote the following:

"Through Time's swift loom our joys and griefs
In braided strands together run,
To weave about this world of ours
Wild tapestries of shade and sun.
And seems it not as if to-night,
Dear, dusty, many-memoried room,
Our souls had lost the threads of light,
And like the eve kept gathering gloom?"

### AMUSEMENTS WITH PROFIT FOR ALL AGES.

**Rules.**—All answers must be addressed to the Prize Editor, The Hospital, 38, Old Jewry, Cheapside, London, E.C., not later than the *first* post on Thursday next. The full name and address must in all cases be given, but a *nom de plume* may be added for publication. Only one side of the paper must be written on.

### FOR MEN AND WOMEN.

**Rule.**—Competitors can enter for *all* quarterly competitions, but no competitor may take more than *two quarterly prizes* during the year.

### Acrostic Competition.

Second quarter commenced February 25th, 1888; ends May 19th, 1888.

A PRIZE of ONE GUINEA will be given to the competitor who gains the highest number of marks for best original acrostics during the ensuing quarter. All acrostics sent in for this competition will be credited according to merit, twelve marks being the highest score given for one acrostic.

A PRIZE of HALF-A-GUINEA to be given to the competitor who gains the highest score for solution of acrostics set. *One mark* only will be given to the competitors who solve all the lights of each acrostic.

Exception will be made in the case of *quotation acrostics*, when *two marks* will be given, *one* for the correct solution of all *lights*, and *one* mark for the *source* of the quotations.

This week credit will be given for solution of

### No. 5.—Double Acrostic.

If in my finals, you my primals
And my finals both can find,
Then in my *primals*, you my *finals*
And my *primals* read combined.

1 "Tongue—doughty giant," dost thou Samson jeer,
Blind, bound, defiant? Venture not too near.

2. First use thine own, use it aright.
Thou'lt soon discern my second light.

3. Faint-hearted oaf, with your cam-coloured beard,
Fair Anne rejects your suit before 'tis heard.

4 Of spirit shrewd, a bride he wooed
Whose temper meant disaster;
But he subdued her haughty mood,
And proved himself the master.

5. If cucumber and lobster
Impair your nightly rest,
Be sure this classic visitor
Will be sitting on your chest.

6. A "'prentice bold" whose feeble frame
Confined rebellion's fiercest flame.

7 To change from hue celestial
To verdant tint of early springtide's bower
Is mine—most magic, and most potent power.

8. Hypothetical and stiff,
The Macedonian phrased his threat;
The threatened people's senate met,
And answered curtly, "If."

### Answers.

No. 4. Double Acrostic

A lembi C

N autc H

Æ theria L

S alammb O

T ige R

H ipp O

E l F

T ors O

I mpropriato R

C ra M

Out of the numerous answers received to the above acrostic, *Dunce* only has succeeded in solving all the lights correctly.

### (A) THE WORD COMPETITION.

Second Quarterly Competition commenced
February 4th, 1888; ends April 28th, 1888.

Three prizes of one guinea 15*s*. and 10*s*. 6*d*. will be given each quarter to the three competitors who obtain the highest number of marks: (1) by making the largest number of words derived from the word or phrase set or anatomical dissection; or (2) for the best answers to (B); or (3) by (1) and (2) combined.

We shall give the newspapers and periodicals for dissection during this quarter, that for this, the *twelfth* week of the quarter, being "The Quiver."

Observe.—Proper names, abbreviations, foreign words, words of less than four letters, and repetitions are barred; plurals, and past and present participles, of verbs are allowed. Nuttall's *Standard* dictionary only to be used.

N.B.—All words sent in must be arranged *alphabetically*, with correct total affixed.

### Results of Tenth Week.

Ellice (44), Patience (42), Butterfly (39), Sym (39), Lauriston (39), Gretta (39), E. E. (38), Lightowlers (38), Brisbane (37), Dunce (34), Nelle (29).

### (B) THE LITERARY COMPETITION.

Some people hate puzzles of all kinds, including word competitions, but they are fond of correspondence, and many sisters and mothers are continually receiving and sending letters to and from members of the family who are abroad, in India or the Colonies. The constant coming and going between England and other portions of the British Empire make it a matter of interest and importance to know something about the life, the climate, the amusements, the adventures, the clothing, and the surroundings of those who seek a livelihood beyond the seas. We shall therefore try to find space for the publication of selected letters from abroad, which may be sent by any of our readers who have friends and relations in foreign lands. We shall give marks for these letters as well as for the word competitions, which will count equally for these prizes, so that anyone may enter for both or either, the prizes to be given to those who get the highest number of marks.

We have received this week a letter for insertion in this column from *Patience*, which is given below.

### Letters from Across the Sea.

Extract of a Letter from New Orleans.

How you would enjoy this city, and the scenery on the Lower Mississippi! I must describe the plan of the city. It is crescent-shaped; the stree through the centre is Canal; on the north side is the French quarter; on the south the American. We are on the American, for which I am thankful. As the city is in form of a crescent, the beautiful simplicity of giving a central street a particular name, and all others at right angles to it, 1st, 2nd, 3rd Street, &c., does not obtain here. In many cases three, and even four, streets open into each other, producing in the mind of the visitor a hopeless confusion. The cars, too, are maddening. If one takes a car in any part of the city out of Canal Street, by the simple plan of staying there long enough you surely land in Canal Street; but take a car there, if you are a visitor, and where you will land has not been recorded. Instance: We wanted to go to Magazine Street; in the rush and confusion took a car on which was printed Upper Magazine, fondly thinking we should have to walk a short distance; on being put down, we found ourselves about four miles out of our way, and had to go back to Canal to get another car. When we reached the place, instead of going about 1½ mile, we had been 9½ miles. Many of the streets here are 4 5, and even 8 miles long. S. Charles' Avenue is one of the best streets; it has beautiful houses on each side of the road for eight miles. Each house stands in beautiful grounds, where even now violets, roses, and camellias are blooming in profusion in the open air; the peach trees and the plum trees are in full bloom, and all the trees are covered with the most delicate green; no word can express the extreme greenness of the grass but the one word "intense." S. Charles' Avenue is about three times wider than North Street, Brighton; in the cent re is the car line; on either side a broad plat of grass, with beautiful shade trees the whole way; on either side of that a wide asphalt road, and then, truth compels me to state, an open drain!—then a wide white pavement. The principal streets on the American side are more or less like this. The houses are provided on north, south, east, and west, with wide piazzas, with the most luxuriant vines, roses, &c., climbing over them. There are many beautiful churches on this side.

*(To be continued.)*

### THE CHILDREN'S CORNER.

PRIZES.
FOR MOST CORRECT ANSWERS TO PUZZLES.
This Commenced October 1st, 1887.
One Pound a Month.
A PRIZE OF THREE GUINEAS
to the Competitor who shall have sent in the largest number of correct or best answers during the twelve months.

### Puzzles—Third Week.

No. 9. Diamond Puzzle.—1. A consonant. 2. One of the prophets. 3. An Indian city. 4. A river in Russia. 5. A province of ancient Greece. 6. A town in Hampshire. 7. A country in Africa. 8. A town in the south of France. 9. A vowel.

a a a a a a a b c c d d d d e e e e e g i i i i m m n n n n n o o o p r r r t v x

No. 10. Charade.

My *first* is a creature that dwells in fresh water,
It's very voracious and much prone to slaughter;
My *second's* an article—'tis not the least,
And my *third* is the plural of some kind of beast;
My *whole* is a frolicsome, mischievous lad,
Somewhat conceited but not really bad.

No 11. Geometrical Puzzle.

Take five pieces of paper of the shape *a*, and five of *b*, and with the ten pieces form a perfect square.

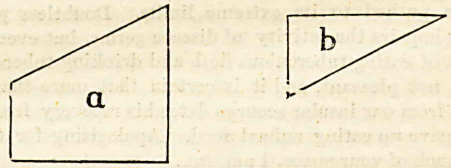

No. 12. Double Acrostic.—1. Something which slaves and felons fear. 2. A large American river. 3. Disorder and hubbub. 4. A fresh-water fish.

The *initials* and *finals* give the title and name of a gallant English admiral.

### Results of First Week's Puzzles.

No. 1. Buried Birds.—1. Swallow. 2. Finch. 3. Emu. 4. Owl.
No. 2. Numerical Puzzle.—COLD.
No. 3. Double Acrostic.

C uf F
R etrieve R
U R I
S wor D
O lympi A
E perna Y

No. 4 Charade. Sigh-Attica = Sciatica.

*All right.*—Nunkey, Plummy Fluff, Bunny, Spider, Scrooge, Good Old Killaloe, McCulloch, Mount Edgecumbe, A. C. K. *Three right.*—Happy Eliza, Judy, Gem. *One right.*—Betty Burke, Poodle.

**Notice to all Correspondents.**—N.B.—All letters referring to this page, which do not arrive at 38, Old Jewry, Cheapside, London, E.C., by the *first post on Thursdays*, and are not addressed PRIZE EDITOR, will in future be disqualified and disregarded.

No one will be permitted to win the Monthly Prizes twice in any one year, the Annual prizes being offered especially to prevent this.

